# B‐cell targeted therapy of pemphigus

**DOI:** 10.1111/1346-8138.16653

**Published:** 2022-12-07

**Authors:** Jun Yamagami

**Affiliations:** ^1^ Department of Dermatology Tokyo Women's Medical University Tokyo Japan

**Keywords:** autoantibody, B cell, Bruton's tyrosine kinase inhibitor, pemphigus, rituximab

## Abstract

Pemphigus is an autoimmune disease that causes blistering and erosion of the skin and mucous membranes because of autoantibodies against desmoglein, which plays an important role in adhesion between epidermal keratinocytes. Treatment of pemphigus has long been centered on corticosteroids, and the guidelines for management of pemphigus have recommended high‐dose systemic corticosteroids as the first‐line treatment. While guideline‐based treatment has been shown to be beneficial in patients with pemphigus, it has also become clear that this treatment is accompanied by significant burden and risk. The challenge for future pemphigus treatment is to maximize efficacy while minimizing risk during the course of the disease. In this regard, treatment targeting B cells is expected to become increasingly important as autoreactive B cells in pemphigus patients are thought to play a major role in the production of autoantibodies, which form the basis of the pathogenesis. The recent expansion of insurance coverage to rituximab, a monoclonal antibody against CD20, for refractory pemphigus in the USA, Europe, and Japan has opened up a new era of pemphigus treatment by enabling treatment strategies with drugs targeting B cells in patients. Here, we discuss the current status and future prospects of pemphigus treatment, focusing on rituximab and Bruton's tyrosine kinase inhibitors, which are expected to become essential drugs for pemphigus treatment in the future.

## 1 INTRODUCTION

Pemphigus is an autoimmune disease that causes blistering and erosion of the skin and mucous membrane due to autoantibodies against desmoglein (Dsg) 1 and 3, components of the desmosomes responsible for epidermal cell adhesion.^1^ Autoreactive B cells are thought to play a major role in the production of autoantibodies.^2^, ^3^ Until recently, treatment of pemphigus was centered on corticosteroids (CS) and other therapies that suppress overall immunity in these patients.^4^, ^5^ With the expansion of insurance coverage to rituximab, a monoclonal antibody against CD20, for refractory pemphigus in the USA, Europe, and Japan, a new era of pemphigus treatment has begun in which therapeutic strategies can be based on drugs targeting B cells in patients. Here, we discuss the current status and future prospects of treatment strategies for pemphigus, focusing mainly on rituximab and Bruton's tyrosine kinase (BTK) inhibitors, which are expected to become essential in the future treatment of this disease.

## 2 GUIDELINE‐BASED TREATMENT OF PEMPHIGUS AND ITS CHALLENGES

**FIGURE 1:**
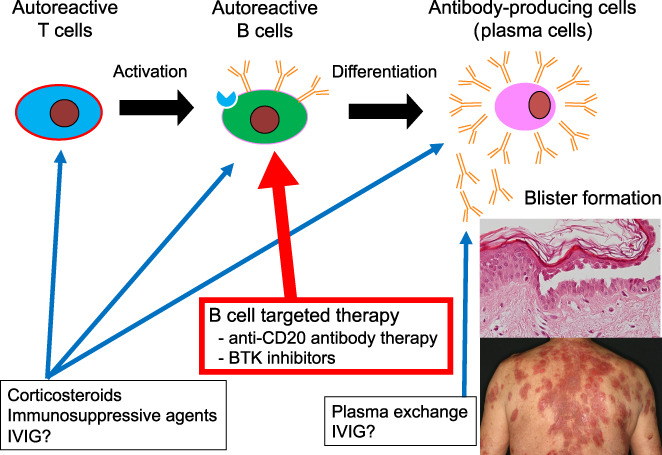
The pathogenesis and targets in the treatment of pemphigus. IVIG, intravenous immunoglobulin.

As pemphigus is an autoimmune disease caused by autoantibodies, the pathogenesis is thought to involve differentiation of autoreactive B cells activated by autoreactive T cells differentiating into plasma cells (antibody‐producing cells), and autoantibodies produced by these plasma cells binding to desmosomes on the surface of epidermal cells and causing blisters (Figure 1). For the dermatologists caring for patients with pemphigus, it is important to understand where the treatment is affecting the disease to develop a treatment strategy. However, pemphigus is a comparatively rare disease, and its treatment is largely dependent on the knowledge and experience of the attending physician. To address this issue, guidelines for management of pemphigus have been published in various countries and regions which define the goal or goals of treatment in the management of pemphigus.^5^, ^6^ The goal of treatment, according to the international definition, is “remission,” i.e., maintenance of pemphigus lesion‐free status with prednisolone (PSL) at ≤0.2 mg/kg/day or 10 mg/day with minimal adjuvant therapy (e.g., immunosuppressive agents).^4^, ^7^ The guidelines emphasize the need to not only to cure the visible blisters and erosions but also to be aware of the possibility of achieving “remission” status in the future. We occasionally encounter cases in which the dose of CS cannot be reduced because the blisters and erosions have not completely disappeared, and such patients are at increased risk of infections, diabetes, osteoporosis, and other side effects associated with long‐term CS administration. Based on the concept that initial treatment should be sufficient to allow the patient to reach “remission” without flare‐ups of symptoms even when the CS dose is reduced, the initial dose of CS recommended by most current guidelines in cases of moderate and severe pemphigus is PSL at 1 mg/kg/day.^4^, ^5^, ^6^

**FIGURE 2:**
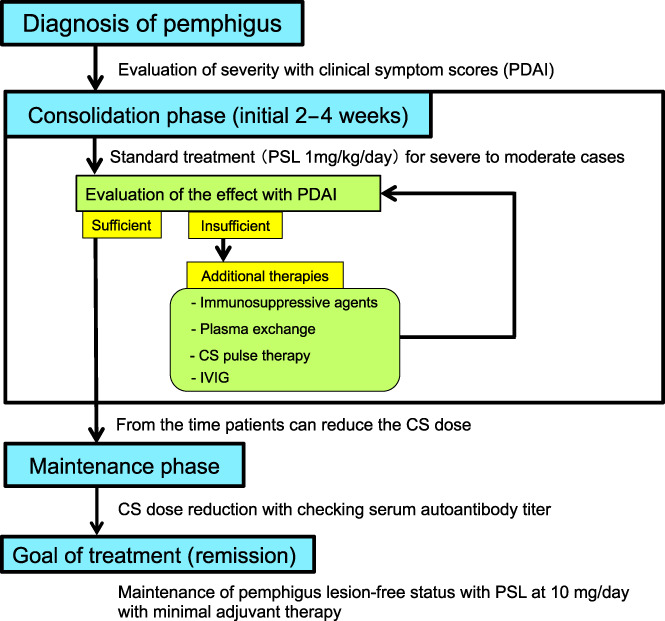
The treatment plan for pemphigus. CS, corticosteroids, IVIG, intravenous immunoglobulin; PDAI, pemphigus disease area index; PSL, prednisolone.

The guidelines recommend that pemphigus treatment should be divided into an induction phase and a maintenance phase (Figure 2).^4^ The induction phase is the period from the start of initial treatment with systemic CS until the disease can be controlled and the CS dose can be reduced, which is approximately 2 weeks after the start of treatment. The goal is to achieve “disease control,” i.e., epithelization of the majority of existing lesions without new blister formation through intensive and adequate treatment.^7^ If the therapeutic effect is judged to be insufficient after 2 weeks, it should be considered whether additional treatment is necessary. Considering the risk of side effects, the same dose of oral CS should not be continued without a clear purpose. As additional treatments, the guidelines list immunosuppressive drugs, CS pulse therapy, plasma exchange therapy, and intravenous immunoglobulin.^4^, ^5^, ^6^ However, considering the mechanism of action of immunosuppressive agents, they may not be suitable for controlling the rapid expansion of blisters and erosions, as it takes more than 1 month for them to reduce autoantibody levels in the patient and thus suppress pemphigus symptoms. Immunosuppressive agents may also be used from the start of treatment, in which case they are not an option for additional treatment.^4^

The treatment maintenance phase is positioned as a period after “disease control” is achieved, during which treatment is maintained with CS reduction. In the maintenance phase, serum autoantibody titers against Dsg, measured by chemiluminescent enzyme immunoassay or enzyme‐linked immunosorbent assay (ELISA), are useful to assess disease activity after clinical symptoms have subsided, and disease activity and serum autoantibody titers basically run in parallel.^8^, ^9^ Once the clinical symptoms have settled and the serum autoantibody titers have decreased or become negative, the CS dose can be reduced with confidence. In some cases, serum autoantibody titers may not become negative even after clinical remission, but they are definitely lower than in the active phase.^10^ In fact, a recent study suggested that the blistering ability of anti‐Dsg autoantibodies is also reduced in response to reduced serum autoantibody titer, suggesting that it is possible to reduce the CS dose while watching for relapse.^11^

To continue to use the optimized guidelines, it is essential to examine treatment outcomes. In a recent study reporting the results of guideline‐based treatment in 84 cases of pemphigus with moderate or severe disease severity, remission was achieved in 83/84 patients (98.8%).^12^ Of the 84 patients enrolled, 53 had pemphigus vulgaris (PV), 28 had pemphigus foliaceus (PF), and three had pemphigus vegetans (PVeg). The mean pemphigus disease area index (PDAI), a clinical symptom score of pemphigus, before treatment initiation was 31.6 (range 9–117). Serum autoantibody titers (ELISA values) to Dsg1 (PF) or Dsg3 (PV and PVeg) before treatment initiation averaged 755.2 (range 23.2–14 993). The initial dose of PSL was 1 mg/kg/day in 77 patients (91.7%) and 0.5 mg/kg/day in seven patients (8.3%), and 73 patients (86.9%) received concomitant immunosuppressive agents (azathioprine in 69 patients and cyclosporine in four patients). The mean time to remission was 13.9 months (range 6.6–84.6 months), and remission was achieved in 58 patients (69.0%) within the first year of treatment and in 78 patients (92.9%) within 2 years. Relapse occurred in 12 patients (14.3%). Adverse events (AEs), including infection (*n* = 56), elevated serum hepatic enzymes (*n* = 38), diabetes (*n* = 19), hyperlipidemia (*n* = 9), hypertension (*n* = 7), decreased white blood cell count (*n* = 7), drug rash (*n* = 7), decreased platelet count (*n* = 5), osteoporosis (*n* = 5), and spinal fracture (*n* = 4), occurred in 67 patients (79.7%) during the treatment period. Grade 3 or higher AEs occurred in 38 patients (45.2%). A 90‐year‐old female patient died of gastrointestinal bleeding during the observation period, but the direct causal relationship to pemphigus and its treatment was unclear. All other AEs were appropriately treated and cured. While the effectiveness of guideline‐based treatment has been demonstrated, it is clear that pemphigus treatment places a great burden and risk on patients.^12^ Future issues, such as devising ways to reduce the frequency of treatment‐related AEs and identifying risk factors in cases that do not achieve remission and in cases of relapse, have been highlighted, with the hope of new therapeutic strategies, such as B‐cell targeted therapy.

## 3 ANTI‐CD20 ANTIBODY THERAPY

**FIGURE 3:**
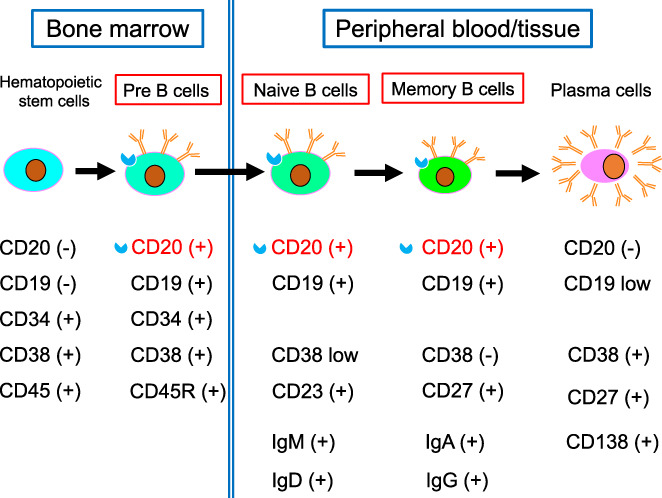
Differentiation of the B‐cell lineage and molecules expressed on the surface. Rituximab selectively eliminates pre‐B cells, naïve B cells, and memory B cells that express CD20 on their surface.

Rituximab (RTX) is an anti‐CD20 human/mouse chimeric antibody that has a human Fc portion and a murine variable region with a CD20 binding site in its large extracellular loop.^13^ CD20 is a transmembrane calcium channel glycoprotein involved in B‐cell activation, proliferation, and differentiation, which is expressed on the cell surface of late pre‐B‐cell stage to naïve follicular B cells and mature memory B cells during B‐cell differentiation (Figure 3).^14^ The binding of RTX to CD20 on the B‐cell surface leads to cell death through a variety of mechanisms, including direct cell death by apoptosis, complement‐dependent cell injury, and Fc‐gamma (Fcγ) receptor‐mediated B‐cell death by cellular effectors, including antibody‐dependent cellular cytotoxicity and antibody‐dependent cell phagocytosis.^15^, ^16^ RTX is considered effective in the treatment of pemphigus because it suppresses autoantibody production by depleting B cells, but its efficacy is thought to depend on a number of factors involved in these different mechanisms, e.g., polymorphisms in the Fcγ receptor.^17^, ^18^ There are two dosing protocols: one for lymphoma treatment, in which a dose of 375 mg/m^2^ is administered four times every week, and the other for rheumatoid arthritis treatment, in which a dose of 1000 mg is administered twice every 2 weeks.^19^, ^20^ The latter appears to be more commonly used recently because it requires fewer infusions and medications, and has been investigated in randomized clinical trials.^21^, ^22^ No trials have compared both protocols, and no significant differences in outcomes between the two regimens have been reported. Reports of cases of refractory pemphigus responding to RTX have been seen since around 2002,^23^ and the results of a prospective study reported in 2007 established the efficacy of RTX.^19^ Although there have not been many prospective studies, more than 500 cases have been reported to date.^24^, ^25^, ^26^ In many patients, new blister and erosion formation ceases and the existing lesions begin to epithelialize, representing a “disease control” condition within 4–6 weeks of commencement of RTX administration. Clinical complete remission (CR) is often achieved within about 6 months after one cycle of RTX treatment in 76%–90% of patients, and 27%–40% of patients have been reported to reach treatment‐free complete remission (CR off therapy) after an average of 15 months. The mean duration of remission is 15–17 months, and it has been reported that CS and immunosuppressive agents can be safely tapered off, although relapses have been reported in about 40% of patients.^27^, ^28^ While safety considerations are important in the administration of RTX, the frequency of serious AEs (SAEs) appears to be lower with its use in autoimmune diseases than in lymphoma.^29^ In practice, it is difficult to determine whether RTX is directly responsible for SAEs in the treatment of pemphigus because it is often given in combination with CS and immunosuppressive agents.^30^ The risk of hematological and infectious events is known to be higher, especially in cases where RTX is combined with other immunosuppressive therapies.^24^, ^29^ RTX‐induced AEs can be divided into short‐term AEs that occur during the infusion or on the following days, and delayed AEs that are mainly related to immunosuppression. Short‐term AEs are characterized by immediate hypersensitivity reactions (hypotension, fever, chills, headache, weakness, nausea, pruritus, urticaria, rash, etc.) associated with infusion‐related reactions (IRRs), i.e., anaphylactic reactions and cytokine release syndrome.^31^ They usually occur more frequently within the first 2 h of infusion, especially with the first two doses. The frequency decreases with subsequent infusions and recovery often occurs without the need for intensive care. In a retrospective study of 92 pemphigus patients, it was reported that IRRs occurred in 61% of cases at the time of the first injection of RTX.^32^ The lower incidence and severity of IRRs compared to B‐cell lymphoma, where IRR occurs in more than 80% of cases, is due to the presumed lower levels of cytokine release associated with RTX administration in pemphigus than in lymphoma.^33^ Although no serious cytokine release syndrome has been reported in pemphigus, one case of death related to acute respiratory distress syndrome has been reported, therefore it is considered necessary to take extra precautions to ensure safety during RTX administration. Another short‐term AE that tends to occur is pemphigus exacerbation.^20^, ^34^ As RTX takes 2–4 weeks to take effect, symptoms may not be controlled in cases of rapidly worsening pemphigus disease activity.^30^ These cases are often predictable because serum autoantibody titers to Dsg are generally elevated, and most cases can be handled with a temporary increase in CS dose.^35^ Most RTX‐related delayed AEs are infections and, moreover, they are the most frequent and most important SAEs for which patients should be monitored. The incidence of infection as a SAE has been variously reported to be up to 40%, with mortality rates of up to 3%.^36^, ^37^ Most infectious SAEs are bacterial infections, often upper/lower respiratory tract or skin infections, and rarely develop into fatal sepsis. On the other hand, a study comparing RTX+CS combination therapy with CS alone reported a higher risk of infection in the CS alone group than in the RTX+CS group.^21^ No increased risk of infection with cumulative dose has been reported in patients receiving multiple doses of RTX, but this will need to be confirmed in long‐term follow‐up studies in the future.^36^

RTX has been used in many countries around the world for the treatment of pemphigus, and has been established itself as a treatment for CS‐resistant cases in several guidelines for the management of pemphigus.^5^, ^38^ Until recently, however, the major problem was that no country has approved RTX treatment for pemphigus as an insurance indication. A major turning point in the expansion of insurance coverage for RTX for pemphigus was the report of a randomized controlled trial of RTX treatment plus short‐term oral CS versus CS monotherapy for initial treatment published in 2017.^21^ In the RTX combination group, 1000 mg/body RTX was administered twice every 2 weeks, followed by an additional 500 mg/body RTX at 12 and 18 months to prevent relapse, and CS was administered at 0.5–1.0 mg/kg/day depending on the severity of the disease and tapered off over 3–6 months. In the CS monotherapy group, CS was started at PSL 1.0–1.5 mg/kg/day and tapered off over 12–18 months. The primary endpoint was the CR rate (no skin or mucosal symptoms without any systemic treatment) after 24 months of RTX treatment, which was 89% (41/46 cases) in the RTX combination group and was significantly better than the rate of 34% (15/44 cases) in the CS monotherapy group. Furthermore, the total CS dose in the RTX combination group was about one third of that in the CS monotherapy group, and side effects were significantly lower. These results led to the approval of an expanded insurance indication for RTX for PV in the USA in 2018 and in Europe in 2019. In terms of comparisons with conventional therapy, the PEMPHIX trial reported in 2021, which compared RTX and mycophenolate mofetil (MMF) in combination with tapered CS therapy in patients with moderate to severe PV, is important.^22^ The primary efficacy endpoint of the study was the percentage of patients who achieved sustained CR (i.e., PDAI of 0 for at least 16 consecutive weeks without the need for systemic administration of CS) during the 52‐week treatment period. The primary endpoint was met in 25/62 patients (40%) in the RTX group, which was significantly higher than the rate of 6/63 patients (10%) in the MMF group. The secondary endpoint, the mean cumulative dose of oral CS during the 52‐week treatment period, was significantly lower in the RTX group than in the MMF group. The incidence of disease flares during the treatment period was significantly lower in the RTX group (5/62 patients, 8%) than in the MMF group (26/63 patients, 41%). AEs occurred in 57/67 patients (85%) in the RTX group and 60/68 patients (88%) in the MMF group. The most frequent AEs in the RTX group were infusion‐related reactions (15 patients, 22%), headache (10 patients, 15%), lymphopenia (eight patients, 12%), and upper respiratory tract infection (eight patients, 12%). SAEs occurred in 15/67 patients (22%) in the RTX group and 10/68 patients (15%) in the MMF group. Serious infections occurred in 6/67 patients (9%) in the RTX group (pneumonia and upper respiratory tract infection, cellulitis and acute pyelonephritis, pyelonephritis, viral pneumonia, infective bursitis, and skin infection) and in 4/68 patients (6%) in the MMF group. All patients with serious infections in each group were treated appropriately and all were cured. No patients in the RTX group had to discontinue treatment or change doses due to serious infections. The results of this trial indicated that RTX is superior to MMF in providing sustained CR and CS reduction for 52 weeks in patients with moderate to severe PV. However, they concluded that more SAEs occurred in the RTX group, and that the accumulation of additional cases and further studies is necessary to assess the long‐term safety and benefit.

In Asia, the efficacy of RTX for pemphigus has been reported in many countries, especially in Korea, China, India, and Singapore.^24^, ^39^, ^40^, ^41^ Although few prospective studies have been conducted, an exploratory study of the efficacy and safety of RTX in CS treatment‐resistant autoimmune bullous diseases was reported in Japan in 2019.^30^ The 10 patients (five men and five women) included in the study consisted of three with PV, six with PF, and one with bullous pemphigoid. The mean age of the patients was 49 years (range 32–73 years), and all were unable to reduce their PSL dose to 10 mg/day due to recurrence. At 40 weeks after RTX administration, five of 10 cases (one PV and four PF) achieved remission. In the remission cases, PDAI was 0 from 4 to 24 weeks after RTX administration, and serum autoantibody titers determined by ELISA had decreased to 25% of the pretreatment level by 6 weeks after commencement of RTX administration. It should be noted that, even in the four pemphigus patients who did not reach remission, both PDAI and serum autoantibody titers improved after PSL was reduced to 10 mg/day. With regard to safety, a total of 58 AEs were reported, 30 of which were infections. Six AEs requiring hospitalization included pneumocystis pneumonia and septic shock associated with pyogenic shoulder arthritis in two patients. All infections were treated appropriately. An important aspect of this study was that both clinical symptom scores and serum autoantibody titers improved with RTX in cases of pemphigus in which remission could not be achieved with conventional therapies. Although the remission rate was somewhat lower than in previous reports, this could be attributed to the fact that “remission” was strictly defined in this study based on PDAI. These results provided the basis for planning a physician‐led clinical trial of RTX for CS treatment‐resistant pemphigus in Japan, and the results of the trial led to approval of expanded coverage of RTX for refractory pemphigus in December 2021.

## 4 BRUTON'S TYROSINE KINASE INHIBITORS

**FIGURE 4:**
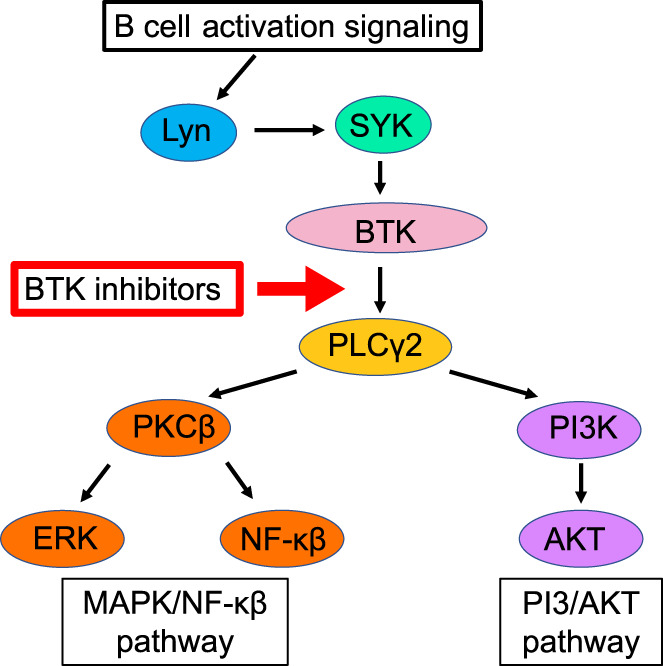
B‐cell activation signaling pathway. BTK inhibitors bind to BTK and inhibit its autophosphorylation, thereby preventing PLCγ2 from being activated and halting the progression of signaling required for B‐cell differentiation and activation. BTK, Bruton's tyrosine kinase; ERK, extracellular signal‐regulated kinase; Lyn, LYN tyrosine kinase; MAPK, mitogen‐activated protein kinase; NF‐κB, nuclear factor‐κB; PI3K, phosphatidylinositol 3‐kinase; PKCβ, protein kinase Cβ; PLCγ2, phospholipase Cγ2

There is a great deal of interest in the application of inhibitors of BTK in pemphigus treatment. BTK is a member of the Tec tyrosine kinase family and is expressed in many hematopoietic cells, including B cells, mast cells, neutrophils, myelocytes, and osteoclasts.^42^ In B cells, BTK is thought to be an important mediator of B‐cell receptor and Fc receptor signaling (Figure 4).^43^ BTK is also known to be involved in B‐cell activation through spontaneous formation of germinal centers, increased production of cytokines, such as interferon‐γ IFN‐γ, interleukin‐6 IL‐6, and interleukin‐1 IL‐1, and expression of costimulatory molecules on B cells.^44^ In addition, BTK mediates many functional responses related to B cell activation, such as maturation, proliferation, antigen presentation, and differentiation into antibody‐producing plasmablasts and plasma cells and, consequently, is involved in the activation of other immune cells that contribute to the pathogenesis of autoimmune diseases, such as T cells and myeloid cells.^45^, ^46^ From the efficacy of RTX as described above, it is clear that B cells are an effective target for pemphigus treatment, and BTK is expected to be an important target for therapeutic interventions without depleting B cells.

Several clinical trials of the BTK inhibitors rilzabrutinib and tirabrutinib for pemphigus have been conducted. Rilzabrutinib is an oral reversible BTK inhibitor that provides increased selectivity and prolonged inhibition due to the formation of both noncovalent and covalent bonds with BTK.^47^ It is also known to have lower cross‐reactivity with other kinases than the first‐ and second‐generation of BTK inhibitors, resulting in lower risks, including off‐target effects.^48^, ^49^ To evaluate the efficacy and safety of rilzabrutinib in pemphigus, a multicenter, proof‐of‐concept, phase II trial (BELIEVE study) was conducted in a number of countries, including Australia, Croatia, France, Greece, and Israel, based on its demonstrated efficacy against canine PF.^50^ Twenty‐seven patients with PV were enrolled and received 400–600 mg/day of rilzabrutinib for 12 weeks. The primary endpoint was the proportion of patients who achieved “control of disease activity” (CDA), defined as the absence of new lesions and beginning of healing of existing lesions within 4 weeks of starting rilzabrutinib with zero or a dose 0.5 mg/kg/day of PSL. Fourteen of the 27 patients (52%) achieved the primary endpoint of CDA with zero‐to‐low dose CS at or prior to week 4, including three patients not initially taking CS. The number of patients who achieved CDA increased over time to 19 cases (70%) at week 12 and 23 cases (85%) after an additional 12 weeks of rilzabrutinib treatment. The combination of rilzabrutinib for 12 weeks successfully reduced the dose of oral steroids in almost all patients. The mean PSL use at week 12 compared to baseline decreased from 20.0 to 11.8 mg/day in newly diagnosed patients and from 10.3 to 7.8 mg/day in patients with relapsed disease. The mean serum autoantibody level against Dsg3 decreased from 404 U/mL at baseline to 289 U/mL after 12 weeks of rilzabrutinib treatment. With regard to safety, 12 of 27 patients (44%) experienced treatment‐related AEs, the most common of which were nausea (15%) and upper abdominal pain (11%), with all others reported by two or fewer patients. Most of these AEs that occurred during the study period were classified as grade 1 or 2 on the Common Terminology Criteria for Adverse Events (CTCAE) and, compared to CS and immunosuppressive agents, there were no significant safety concerns associated with rilzabrutinib. Unfortunately, the subsequent phase 3 PEGASUS trial failed to meet its primary endpoint, raising concerns about the future of development of rilzabrutinib in pemphigus. As the phase 2 BELIEVE showed promising results, many patients and stakeholders wish to see development continue.

Tirabrutinib is another highly selective oral BTK inhibitor, which has been approved in Japan for the treatment of central nervous system primary lymphoma, primary macroglobulinemia, and lymphoplasmacytic lymphoma.^51^, ^52^, ^53^ Tirabrutinib has been reported to inhibit stimulation‐induced IgG production in human B cells and prevent further elevation of anti‐double‐stranded DNA antibody levels in spontaneous lupus‐prone mice.^54^ A clinical trial of 80 mg/day tirabrutinib for 52 consecutive weeks was conducted in Japan in patients with pemphigus refractory to CS therapy.^55^ The primary endpoint was the proportion of patients in CR (pemphigus symptom‐free for 8 weeks with oral CS ≤10 mg/day PSL equivalent and minimal concomitant therapy) after 24 weeks of tirabrutinib therapy, which was achieved in 3/16 patients (18.8%) who entered the study. Interestingly, the CR rate up to 52 weeks after initiation of tirabrutinib was 50% (8/16 cases), and the cumulative remission rate, including partial remission (near CR but with the appearance of pemphigus lesions that healed spontaneously within 1 week) was 62.5% (10/16 cases). The oral dose of PSL decreased from 17 mg/day before initiation of tirabrutinib to 7.6 mg/day after 52 weeks. These results suggest that tirabrutinib tends to take somewhat longer to show its effects. AEs during the trial occurred in 14/16 patients (87.5%), with multiple cases of nasopharyngitis (five cases), influenza (three cases), pemphigus exacerbation (three cases), hypertension (three cases), folliculitis (two cases), oral candidiasis (two cases), and increased hepatic enzyme levels (two cases), the majority of which were CTCAE grade 1 or 2. Grade 3 AEs were reported in 4/16 patients (25%), including pemphigus exacerbation in two cases and pharyngitis, hypertension, stroke, steroid diabetes, gastric cancer, and elevated blood alkaline phosphatase level in one case each. No grade 4 AEs were reported. All serious and grade 3 AEs were ruled out as being associated with tirabrutinib. AEs that resulted in discontinuation of treatment were observed in 2/16 patients (12.5%), including pemphigus exacerbation (one case) and gastric cancer (one case), both of which were determined to be unrelated to tirabrutinib. Based on these results, it is unlikely that there are significant safety concerns compared to conventional pemphigus treatments. Focusing on B cells and antibody‐producing functions, the mean percentage changes from baseline in serum autoantibodies to Dsg1 and Dsg3 were −55.8% and −39.4% at week 8 and −89.3% and −60.4% at week 52, respectively. On the other hand, the mean percentage changes from baseline (before tirabrutinib) after 52 weeks of treatment were −4.2% for serum total IgG and −10.5% for CD19‐positive B‐cell counts in the peripheral blood. These results are in contrast to the observation that no B cells were detected in the peripheral blood of patients treated with RTX for more than 1 year. Presumably, tirabrutinib inhibits B‐cell development and activation by blocking BTK in the B‐cell receptor cascade without depleting B cells for a long period, unlike RTX (Table 1). Tirabrutinib was reported to have a half‐life of 6.5–8 h, suggesting that its pharmacological effects disappear rapidly.^56^ This may be advantageous in that SAEs, such as infections, can be treated promptly during the course of treatment. The convenience of a once‐daily oral formulation also suggests that tirabrutinib may reduce the physical and emotional burden experienced by pemphigus patients and, although its effect on pemphigus may be milder than that of RTX, its continued development is expected in the future.

**TABLE 1:** Comparison of rituximab and BTK inhibitors in the treatment of pemphigus

	Rituximab	BTK inhibitors
Serum autoantibody levels to Dsg	Dramatic decrease after 2–4 weeks of treatment The effect peaks after 12–20 weeks of treatment, with a gradual increase, especially in relapsing cases	Decrease after 2–4 weeks of treatment The effect lasts during oral administration
Serum total IgG levels	Tendancy to decrease up to 12 weeks after treatment, with a maximum decrease of 50% in some cases Return to pre‐treatment levels after 6 months of treatment in many cases	Decrease slightly after 4–12 weeks of treatment, but recover to pre‐treatment levels even with continued oral administration
B cell counts in the peripheral blood	Below the detection limit immediately after administration B cells are not detected in the peripheral blood for more than a year after administration in many cases	Little or no decrease even with continued oral administration

Abbreviation: BTK, Bruton's tyrosine kinase; Dsg, desmoglein.

## 5 CONCLUSION

The challenge for the future of pemphigus treatment is to elicit high efficacy with a lower risk of AEs during the course of treatment. In this respect, B‐cell targeted therapies will become increasingly important. It is hoped that the ideal use of RTX for refractory cases will be established, as well as the continued development of BTK inhibitors and the next generation of anti‐CD20 therapy.

## CONFLICT OF INTEREST

The author has received research funding, consultancy fees, lecture fees, and travel expenses from Zenyaku Kogyo Co. Ltd., and Ono Pharmaceutical Co., Ltd.
